# Imaging of compartmentalised intracellular nitric oxide, induced during bacterial phagocytosis, using a metalloprotein–gold nanoparticle conjugate†
†Electronic supplementary information (ESI) available: Experimental procedures and additional experimental data (Fig. S1–S7 and Tables S1–S3) (PDF). See DOI: 10.1039/c7an00898h


**DOI:** 10.1039/c7an00898h

**Published:** 2017-09-29

**Authors:** Richard Leggett, Paul Thomas, María J. Marín, Jelena Gavrilovic, David A. Russell

**Affiliations:** a School of Chemistry , University of East Anglia , Norwich Research Park , Norwich , Norfolk NR4 7TJ , UK . Email: d.russell@uea.ac.uk; b School of Biological Sciences , University of East Anglia , Norwich Research Park , Norwich , Norfolk NR4 7TJ , UK . Email: j.gavrilovic@uea.ac.uk

## Abstract

Nitric oxide (NO) plays an essential role within the immune system since it is involved in the break-down of infectious agents such as viruses and bacteria. The ability to measure the presence of NO in the intracellular environment would provide a greater understanding of the pathophysiological mechanism of this important molecule. Here we report the detection of NO from the intracellular phagolysosome using a fluorescently tagged metalloprotein–gold nanoparticle conjugate. The metalloprotein cytochrome *c*, fluorescently tagged with an Alexa Fluor dye, was self-assembled onto gold nanoparticles to produce a NO specific nanobiosensor. Upon binding of NO, the cytochrome *c* protein changes conformation which induces an increase of fluorescence intensity of the tagged protein proportional to the NO concentration. The nanobiosensor was sensitive to NO in a reversible and selective manner, and exhibited a linear response at NO concentrations between 1 and 300 μM. In RAW264.7γ NO^–^ macrophage cells, the nanobiosensor was used to detect the presence of NO that had been endogenously generated upon stimulation of the cells with interferon-γ and lipopolysaccharide, or spontaneously released following treatment of the cells with a NO donor. Significantly, the nanobiosensor was shown to be taken up by the macrophages within phagolysosomes, *i.e.*, the precise location where the NO, together with other species, destroys bacterial infection. The nanobiosensor measured, for the first time, increasing concentrations of NO produced during combined stimulation and phagocytosis of *Escherichia coli* bacteria from within localised intracellular phagolysosomes, a key part of the immune system.

## Abstract 2

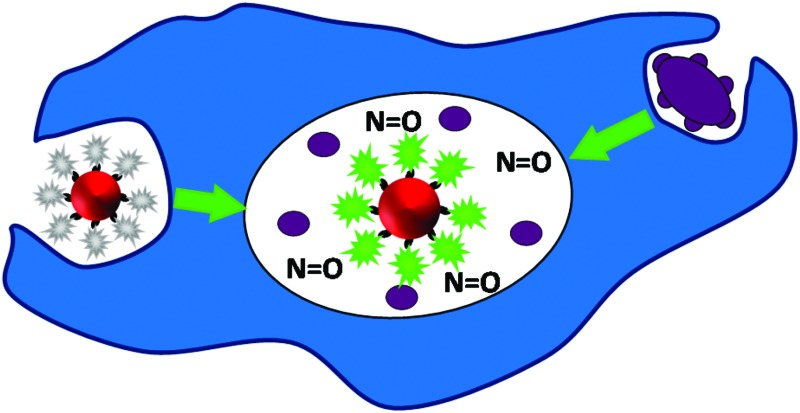
Imaging of the *in situ* production of nitric oxide following phagocytosis of *Escherichia coli* bacteria using a NO nanobiosensor.

## Introduction

Nitric oxide (NO) is a gaseous free radical that plays an important role in the regulation of diverse physiological and pathophysiological mechanisms of the cardiovascular, nervous and immune systems.^1^,^2^ In mammalian cells, NO is produced by the NO synthase (NOS) enzymes, specifically, neuronal NOS (nNOS), endothelial NOS (eNOS) and inducible NOS (iNOS).^3^,^4^ The NO generated in the brain by nNOS acts as a neuromediator to influence functions such as behaviour and memory; smooth muscle control and gastrointestinal motility are influenced by the NO generated in the peripheral nervous system where the molecule acts as a neurotransmitter.^5^ Inappropriate regulation of nNOS has been implicated in a number of neurodegenerative diseases^6^ such as Huntington's^7^ and Parkinson's^8^ diseases. NO produced by the eNOS of endothelial cells functions as a vasodilator thereby regulating blood flow and pressure.^9^ In macrophages, infectious agents, such as bacteria or viruses, are phagocytosed and ultimately destroyed by the production of NO by iNOS (also referred to as NOS2).^10^ The phagosome containing the infectious agent matures and ultimately fuses with lysosomes to form the phagolysosome where the low values of pH, the presence of lysosomal enzymes, and the production of NO and reactive oxygen species provide an ideal environment for the breakdown of the infectious agents.^11^

With consideration of the significant roles of NO, the development of sensitive and selective methods to detect and quantify intracellular NO in a localised and real-time manner is essential. Several methodologies currently exist for the study of intracellular NO that are based on chemiluminescence, electrochemical, electron paramagnetic resonance (EPR) or fluorescence methods.^12^ In particular, fluorescence based organic and inorganic molecules have been synthesised to image intracellular NO and have already provided considerable insight into the role that NO plays in biology.^12^–^20^ A lysosome-targetable multifunctional probe, based on the intramolecular luminescence resonance energy transfer from a Tb^3+^ complex to a rhodamine derivative, has been reported recently for the ratiometric and lifetime detection of NO *in vitro* and *in vivo* with a limit of detection of 1.8 μM.^21^ Eroglu *et al.* have developed genetically encoded fluorescent probes to image subcellular NO dynamics in real-time.^22^ These fluorescent probes, derived from bacterial NO-binding domains, were able to detect NO concentrations as low as 50 nM. In addition, there have been some recent reports of nanosensors and nanoprobes for the intracellular imaging and sensing of NO.^23^–^27^ Of particular relevance for the measurement of NO are: the functionalised gold nanoparticles encapsulated in a silica capsule used for Surface Enhanced Raman Spectroscopy (SERS) detection^25^ and; the rhodamine B derivative used to functionalise the pores of mesoporous silica nanoparticles for fluorescence based detection in living cells and in a mouse model.^26^ Both of these nanoprobes have been used to detect nanomolar concentrations of NO from within lysosomes of cells. However, to the best of our knowledge, no nanoparticle based system has reported the *in situ* production of compartmentalised NO during bacterial phagocytosis. Such visualisation of the real-time production of NO would be a powerful tool for elucidating the biological role that NO plays in the destruction of infectious agents such as bacteria.

**Fig. 1:**
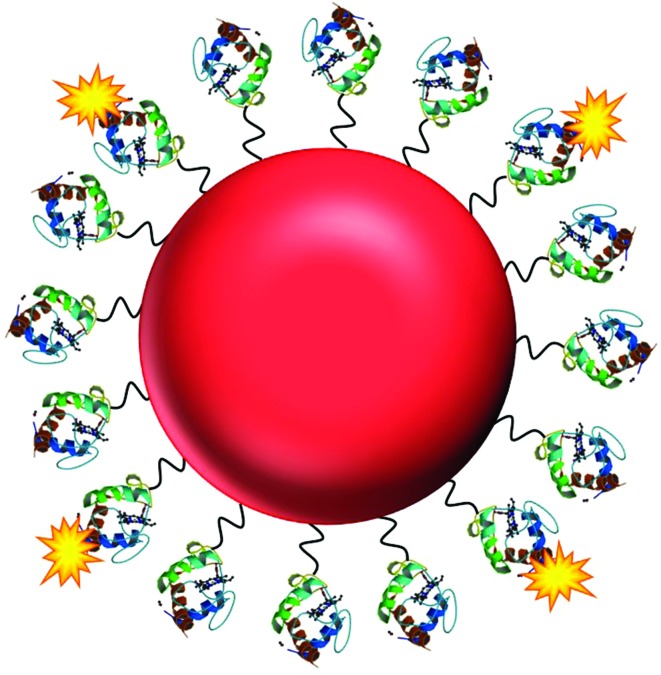
The NO nanobiosensor: cytochrome *c* (structure obtained from the Protein Data Bank – PDB ID ; 1HRC)^28^ fluorescently tagged with Alexa Fluor 488 (yellow) assembled onto a gold nanoparticle (red) surface *via* a SPDP linker (black).

Here, we present the development of a fluorescence based NO nanobiosensor using gold nanoparticles functionalised with fluorescently tagged cytochrome *c* metalloproteins (Fig. 1) which is capable of detecting NO in a reversible and selective manner. Cytochrome *c* was chosen as the biological recognition molecule since the iron containing porphyrin prosthetic group will selectively bind NO following displacement of the proximal methionine ligand.^29^,^30^ The displacement of the methionine amino acid by the NO molecule, induces a conformational change within the cytochrome *c* protein. By fluorescently tagging the cytochrome *c* on the gold nanoparticle, the change in conformation of the protein actuates an increase in the fluorescence intensity of the conjugates that is directly proportional to the concentration of the NO. The cytochrome *c* – gold nanoparticle conjugates were used to detect NO from the precise organelles within RAW264.7γ NO^–^ macrophages where the NO is located. Significantly, the nanoconjugates were used to image the *in situ* production of NO induced in the phagolysosomes within macrophage cells during a combined stimulation and phagocytosis of *Escherichia coli* (*E. coli*) bacteria.

## Results and discussion

The haem centre of cytochrome *c* is known to bind NO following displacement of the methionine ligand with a consequent change in its UV-visible absorption spectrum.^29^,^30^ However, the change in the absorption spectrum is insufficiently sensitive to measure changes of NO at the concentrations found within the intracellular environment. Consequently, purified cytochrome *c* was tagged with a fluorescent Alexa Fluor 488 (a488) derivative, covalently attached *via* the lysine residues on the protein surface. The modification of cytochrome *c* with the a488 was confirmed by the fluorescence emission spectrum of the protein before and after modification (ESI, Fig. S1†). By fluorescently tagging the cytochrome *c*, the change of the protein conformation following NO binding resulted in a change in the fluorescence intensity of the metalloprotein directly proportional to the concentration of the NO. The fluorescently tagged protein was then further functionalised with *N*-succinimidyl 3(2-pyridyldithio)-propionate (SPDP) to provide a disulphide moiety with which to self-assemble the protein to the nanoparticle surface through a sulphur–gold bond. The functionalisation of the cytochrome *c*-a488 complex with SPDP was confirmed using dl-dithiothreitol (DTT).^31^ The importance of the cytochrome *c* purification, the cytochrome *c* to fluorophore molar ratio (4 : 1 as shown in Fig. S2†), and the order of functionalisation of the protein with both a488 and SPDP were all investigated to achieve the optimised NO nanobiosensor (see Experimental section). Gold nanoparticles (*ca.* 16 nm) were prepared using a modification^32^ of the Enüstün and Turkevich method^33^ whereby hydrogen tetrachloroaurate was reduced by sodium citrate. The NO nanobiosensor was constructed by mixing the SPDP-cytochrome *c*-a488 complex with a 3 nM solution of the gold nanoparticles for 48 h. The optimum ratio of SPDP-cytochrome *c*-a488 complex to stabilise the nanoparticles was estimated to be 120 : 1 as determined using a flocculation assay.^34^ The fluorescently tagged cytochrome *c* nanoparticle conjugates were purified using 30 kDa MW cut-off centrifuge tubes.

**Fig. 2:**
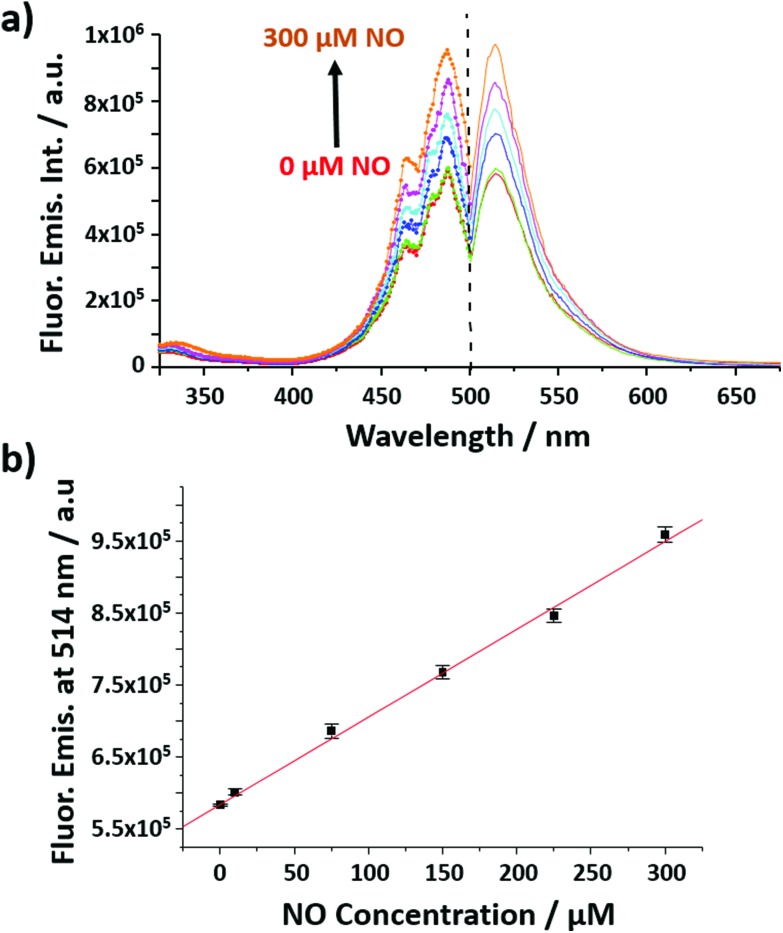
(a) Fluorescence excitation (left; *λ*_ems_ = 514 nm) and emission (right; *λ*_exc_ = 492 nm) spectra of the NO nanobiosensor in the presence of increasing concentrations of NO (from 0 to 300 μM). (b) Calibration curve of the NO nanobiosensor obtained from the fluorescence emission intensity at 514 nm as a function of the concentrations of NO; error bars show the mean error of 3 replicates. *y* = 1217*x* + 584 141.

The nanobiosensor (Fig. 1) was calibrated with gaseous solutions of NO in an oxygen free environment. An increase in both the fluorescence excitation and emission intensities of the NO nanobiosensor was observed with increasing concentration of NO from 1 to 300 μM (Fig. 2). To investigate the selectivity of the nanobiosensor towards NO, a number of potential interferences were studied. The interferences were chosen based on two criteria: (1) species possibly found within the macrophage cells such as hydrogen peroxide, superoxide radical anion, peroxynitrite anion, nitrite and nitrate; and (2) reagents used during the cell culture procedures (Table S1†). The interferences were added to the NO nanobiosensor in the absence and presence of NO (40 μM) and the fluorescence emission spectra recorded. A fluorescence intensity deviation by >2% from that of the control was judged to be a significant interference. Of the possible interferences examined (Fig. S3†), a pH of 4 produced a decrease in fluorescence intensity of 4% in the presence of NO. The pH of the acidic organelles in macrophages is typically 4.0–5.5.^35^,^36^ The interference effect at pH < 4 would be to reduce the sensitivity of the NO nanobiosensor rather than produce a false positive result. A 5 μM superoxide radical anion concentration increased the fluorescence intensity by 3% in the absence of NO. This concentration is far in excess of that typically observed in macrophages.^37^ Therefore, it was determined that neither of these interferences were likely to be significant within the cells being measured. The NO nanobiosensor was fully reversible as determined by five sequential cycles of addition and removal of NO (Fig. S4†). In addition, the NO nanobiosensor was stable in solution for 4 days, maintaining its limit of detection for NO of *ca*. 2 μM. For the work reported here, the NO nanobiosensor was freshly synthesised and calibrated prior to each experiment.

**Fig. 3:**
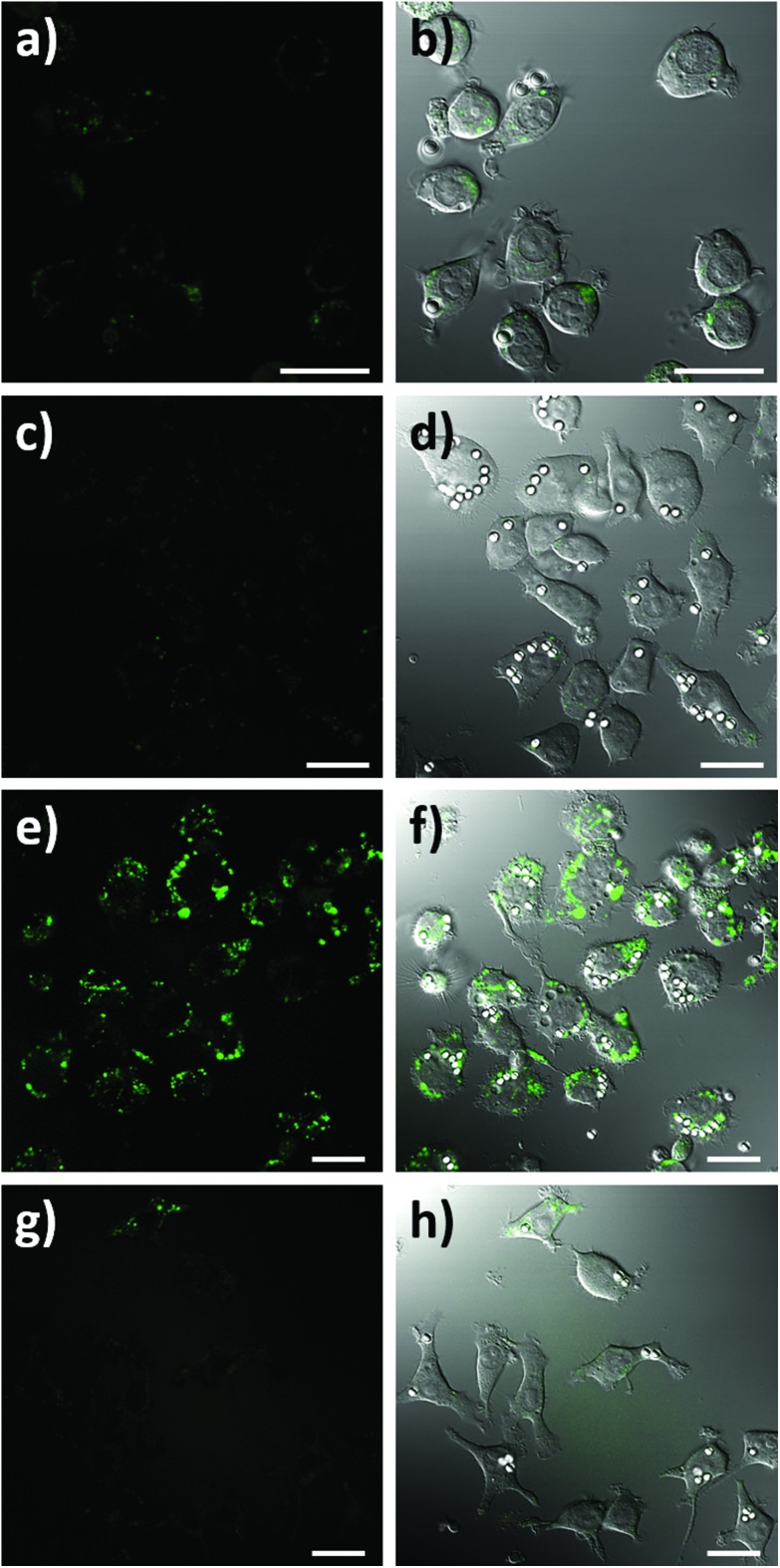
Confocal fluorescence microscopy images of RAW264.7γ NO^–^ cells incubated overnight with the NO nanobiosensor (10 nM) under different conditions: (a and b) unstimulated control cells; (c and d) stimulated with INF-γ (10 ng mL^–1^); (e and f) stimulated with INF-γ (10 ng mL^–1^) and LPS (500 ng mL^–1^); and (g and h) stimulated with INF-γ and LPS in the presence of l-NAME. The cells were challenged for 1 h with latex beads (white) prior to imaging. Images a, c, e and g are fluorescence images (green channel, 505–545 nm; *λ*_ex_ = 488 nm). Images b, d, f, and h are composite images of the green and differential interference contrast (DIC) channels. Scale bars = 20 μm.

To assess the intracellular NO sensing potential of the nanobiosensor, the mouse macrophage cell line RAW264.7γ NO^–^ was used. Macrophages are integral to the immune response and when activated by a foreign body increase their production of NO.^38^ The external stimulation of RAW264.7γ NO^–^ cells to produce NO requires both interferon-γ (IFN-γ) and lipopolysaccharide (LPS) for full activation, making its behaviour more typical of primary macrophages.^39^ The sensing capability of the NO nanobiosensor was evaluated using cells that had been incubated overnight with the nanobiosensor and treated under four different stimulation conditions to produce varying amounts of NO: (1) unstimulated (control) cells; (2) stimulated with IFN-γ alone; (3) stimulated with both IFN-γ and LPS; and (4) stimulated with both IFN-γ and LPS together with an inhibitor of iNOS, *N*_ω_-nitro-l-arginine methyl ester hydrochloride (l-NAME).^40^ To mimic the uptake of foreign bodies and to further challenge the macrophage cells, 3 μm latex beads were added and incubated for 1 h prior to imaging. As seen from the confocal fluorescence microscopy images in Fig. 3, the NO nanobiosensor was taken up by the RAW264.7γ NO^–^ cells in all instances, although varying fluorescence emission intensities were observed dependent on the stimulation conditions used. The fluorescence emission intensity was low in both the control, unstimulated cells (Fig. 3a and b) and cells that had been stimulated with IFN-γ alone (Fig. 3c and d) due to the reduced levels of NO present in these cells as detected by the nanobiosensor. When the cells had been stimulated with both IFN-γ and LPS, a substantial increase in the fluorescence emission intensity was observed indicating an increase in the production of NO as detected by the nanobiosensor (Fig. 3e and f). The increase in the fluorescence emission intensity of the NO nanobiosensor was not observed when the cells had been stimulated with IFN-γ and LPS in the presence of l-NAME (Fig. 3g and h). This latter result confirms that, under these conditions, the production of NO was significantly reduced due to the inhibition of iNOS, and further shows that the nanobiosensor reports on the specific production of intracellular NO. The fluorescence emission intensities of the NO nanobiosensor within the macrophage cells under the four stimulation conditions were measured and the results, with their statistical analysis, are reported in Table S2.† These results highlight the detection of NO when the cells are stimulated with both INF-γ and LPS as shown in the confocal images of Fig. 3.

**Fig. 4:**
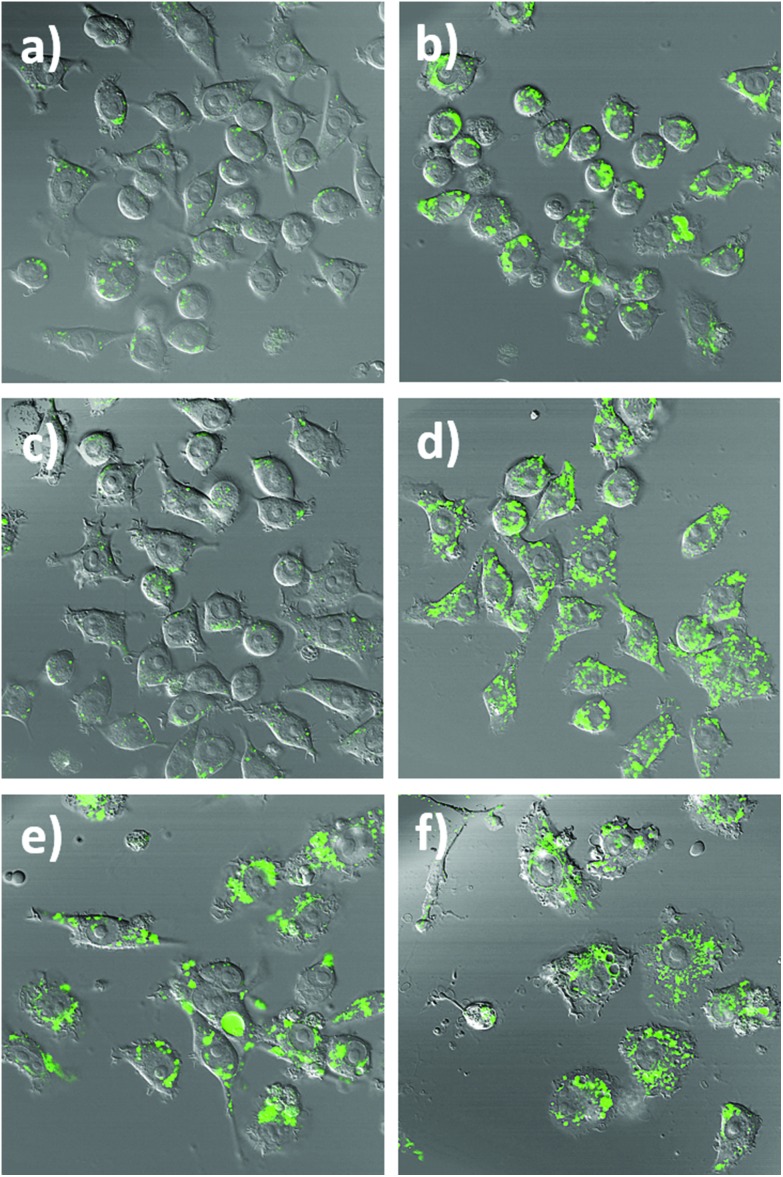
Confocal fluorescence microscopy images of RAW264.7γ NO^–^ cells incubated with the NO nanobiosensor (10 nM) under different conditions: (a and b) control, (c and d) IFN-γ (10 ng mL^–1^) only, and (e and f) IFN-γ (10 ng mL^–1^) and LPS (500 ng mL^–1^) before (a, c and e) and after (b, d and f) the addition of the NO donor SNAP (100 μM). The green fluorescence is due to the emission of the NO nanobiosensor upon excitation at 488 nm (emission collected between 505–545 nm). Images (a)–(f) are composite images of the green and DIC channels.

To further highlight the intracellular NO sensing capability of the nanobiosensor, RAW264.7γ NO^–^ cells (Fig. 4 and S5†) that had been incubated overnight with the nanobiosensor and stimulated under different conditions (IFN-γ only; INF-γ and LPS; and unstimulated) were treated with the NO donor *S*-nitroso-*N*-acetylpenicillamine (SNAP)^41^ 40 min prior to imaging with the confocal microscope. For the unstimulated cells and the cells stimulated with IFN-γ only, a dramatic increase of the fluorescence emission intensity from the NO nanobiosensor in specific regions within the intracellular environment was observed following incubation with SNAP (Fig. 4a–d and S4†). For the cells stimulated with both INF-γ and LPS the fluorescence intensity of the NO nanobiosensor was similar before or after addition of the SNAP (Fig. 4e and f). These results confirm the ability of the nanobiosensor to report the presence of intracellular NO and show that the take-up of the NO nanobiosensor by the RAW264.7γ NO^–^ cells was independent of the stimulation conditions. The fluorescence emission intensities of the cells in the absence and in the presence of the NO donor SNAP were measured. These fluorescence intensity values and the statistical analysis are shown in Table S3.†

**Fig. 5:**
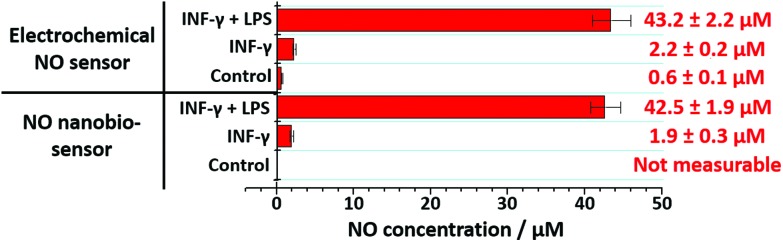
Extracellular measurements of NO. The NO concentration present in the supernatant of RAW264.7γ NO^–^ macrophage cells growth medium was assessed using the NO nanobiosensor and compared with a commercial electrochemical sensor.

The nanobiosensor was subsequently used to quantify the NO produced by the RAW264.7γ NO^–^ macrophage cells in the extracellular environment. These measurements were then compared to those obtained using a commercial electrochemical sensor. The NO concentration in the extracellular medium was measured for non-stimulated RAW264.7γ NO^–^ cells and those cells that had been stimulated overnight with either INF-γ only, or with INF-γ and LPS. In an oxygen free environment, the concentrations of NO determined using the NO nanobiosensor were found to be in agreement with those determined using the electrochemical NO sensor (Fig. 5). Based on the studies of extracellular NO, a limit of detection of *ca.* 2 μM was estimated for the NO nanobiosensor.

**Fig. 6:**
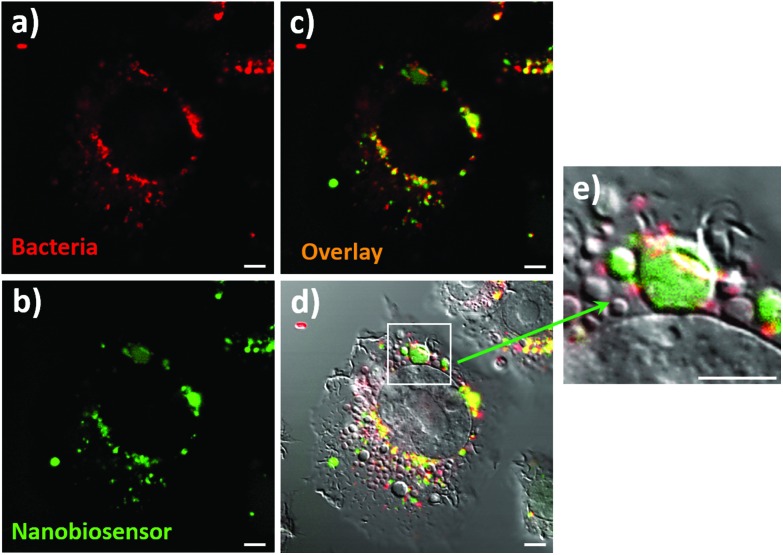
Confocal fluorescence microscopy images of RAW264.7γ NO^–^ cells stimulated with IFN-γ (10 ng mL^–1^) and LPS (500 ng mL^–1^) and incubated with the NO nanobiosensor (10 nM) overnight; and then challenged with Texas Red stained *E. coli* bacteria for 3 h prior to imaging. Fluorescence images collected in: (a) red channel (560–750 nm, *λ*_exc_ = 543 nm) and (b) green channel (505–545 nm, *λ*_exc_ = 488 nm). (c) and (d) Are composite images of the red and green; and the red, green and DIC channels, respectively. (e) Is a magnified image of the phagolysosome shown in (d). Scale bars = 5 μm.

The ultimate goal of this work was the detection of compartmentalised intracellular NO produced by RAW264.7γ NO^–^ cells during bacterial phagocytosis. To be able to achieve this goal, both the NO nanobiosensor and the engulfed bacteria should co-localise within intracellular compartments of RAW264.7γ NO^–^ macrophage cells that had been challenged with a bacterial infection. Denatured *E. coli* bacteria stained with Texas Red (to enable fluorescence imaging) were added to RAW264.7γ NO^–^ cells that had been stimulated overnight with IFN-γ and LPS and incubated with the NO nanobiosensor (Fig. 6). The Texas Red stained *E. coli* were phagocytosed by the RAW264.7γ NO^–^ cells within 2 to 4 h. When incubated for 3 h, intact or fragmented phagocytosed *E. coli* bacteria were observed in the macrophage cells (Fig. S6†). The red emission of the Texas Red labelled *E. coli* bacteria (Fig. 6a) co-localises with the green emission of the NO nanobiosensor (Fig. 6b) inside the stimulated RAW264.7γ NO^–^ cells as highlighted by the yellow coloration shown in the overlay image (Fig. 6c). The combined differential interference contrast (DIC), red and green channels (Fig. 6d) highlights the compartmentalised nature of the NO sensing using the NO nanobiosensor within the intracellular environment. The magnified image (Fig. 6e) shows the typical elongated structure of an *E. coli* bacterium co-localised with the NO nanobiosensor, both contained within a putative phagolysosome.

**Fig. 7:**
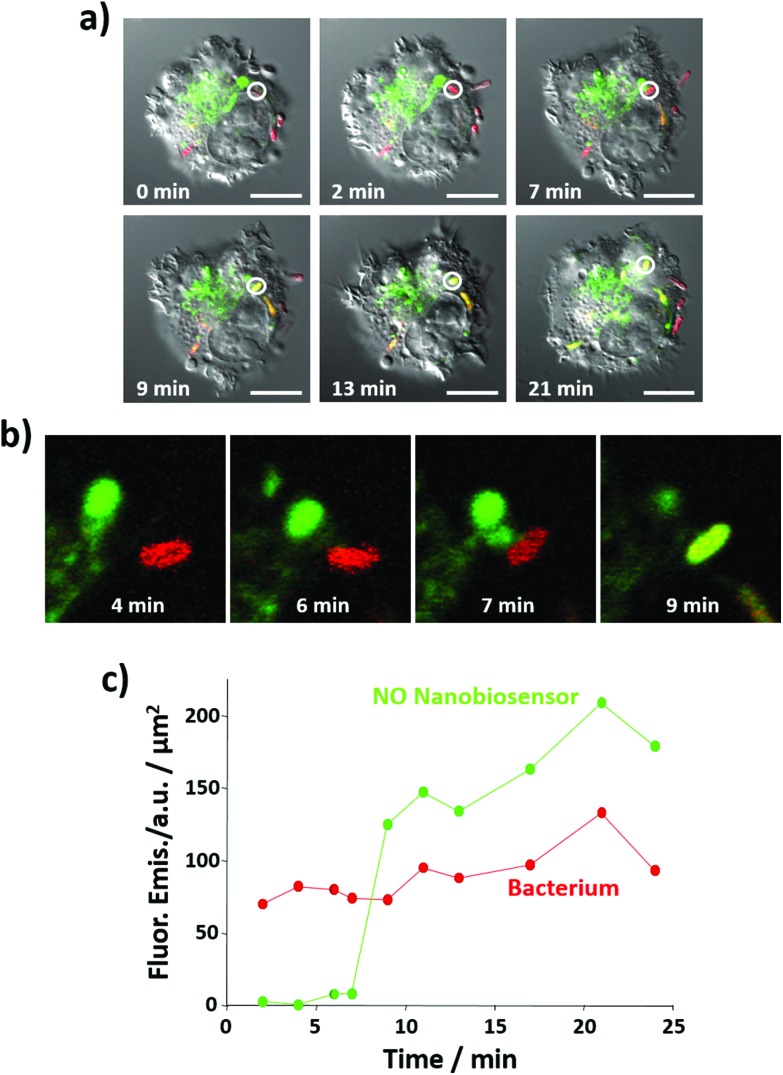
Time course of NO generation during bacterial phagocytosis as measured by the NO nanobiosensor. (a) Confocal fluorescence microscopy images of live RAW264.7γ NO^–^ cells stimulated with IFN-γ (10 ng mL^–1^) and LPS (500 ng mL^–1^) and incubated with the NO nanobiosensor (10 nM) overnight, and then challenged with Texas Red stained *E. coli* bacteria for 3 h prior to imaging. The images, taken at t = 0, 2, 7, 9, 13 and 21 min, are composite images of the green (NO nanobiosensor, *λ*_exc_ = 488 nm, emission collected between 505 and 545 nm), red (Texas Red tagged *E. coli*, *λ*_exc_ = 543 nm, emission collected between 560 and 750 nm) and DIC channels (*λ*_exc_ = 488 nm). Images were captured using the ‘line by line’ function of a Zeiss LSM 510 confocal microscope. The white circle indicates a region of interest where an *E. coli* fragment co-localises with the NO nanobiosensor at *t* = 9 min. Scale bars = 10 μm. (b) Magnified image of the region where the NO nanobiosensor co-localises with the *E. coli* fragment in (a) at *t* = 4, 6, 7 and 9 min. (c) Fluorescence emission intensity, plotted as the mean value per unit area, of the NO nanobiosensor (green) and the Texas Red tagged *E. coli* (red) as a function of time.

Importantly, the NO nanobiosensor was used to monitor the *in situ* production of intracellular NO following combined stimulation and bacterial phagocytosis. The uptake of *E. coli* bacteria by the RAW264.7γ NO^–^ cells, and the distribution of the fluorescence emission from the NO nanobiosensor were monitored using time-lapse confocal microscopy by taking a fluorescence image *ca.* every 2 min for a period of 24 min (Fig. 7a, b and S7†). The fluorescence images were complemented with measurements of the fluorescence emission intensity per μm^2^ of the NO nanobiosensor and the Texas Red labelled *E. coli* (Fig. 7c). At 2 min, an *E. coli* bacterium had been phagocytosed by the macrophage cell as observed by the red fluorescence from the labelled bacterium (white circle in Fig. 7a – 2 min). After 9 min, some of the NO nanobiosensors had co-localised with the bacterium as indicated by the yellow overlay colour observed in the white circle in Fig. 7a – 9 min. At this time interval, it is possible that the vacuole containing the NO nanobiosensor (lysosome) and the vacuole containing the bacterium (phagosome) fuse and form a phagolysosome. The merging of the two vacuoles can be seen in the magnified images shown in Fig. 7b. In the phagolysosomes, NO, together with other species, induces the degradation of bacteria. The presence of NO in the vacuole, where both the nanobiosensor and the bacterium co-localised, was confirmed by the measurements of the fluorescence intensity shown in Fig. 7c. Following co-localisation at 9 min, a steady increase in the fluorescence intensity from the NO nanobiosensor was observed up to 24 min. Such an increase of fluorescence intensity would be expected with increasing NO concentration within the macrophage during bacterial phagocytosis. With consideration of the limit of detection, it is apparent from Fig. 6 and 7 that the concentration of NO in the phagolysosome is at least 2 μM and, probably, significantly higher than this lower limit. This result is consistent with the measurement of *ca*. 8 μM NO obtained using a fluorescence based rhodamine-silica nanoparticle probe.^26^

## Conclusions

A metalloprotein–gold nanoparticle conjugate has been synthesised based on fluorescently tagged cytochrome *c* self-assembled to gold nanoparticles *via* a SPDP linker to form a NO sensitive nanobiosensor. The NO nanobiosensor was selective, reversible, provided a linear response between 1–300 μM and exhibited a limit of detection of *ca.* 2 μM. The NO nanobiosensor was used to image the localised intracellular production of NO in macrophages. Morphological or internal compartmentalisation differences between cells treated with or without the NO nanobiosensor were not observed suggesting that the nanobiosensor does not compromise cellular activity. Importantly, the nanobiosensor was shown to monitor the increasing concentrations of NO produced during bacterial phagocytosis from within localised compartments of the macrophage cells. The ability to measure NO from within specific intracellular organelles using the fluorescence based nanobiosensor provides an important additional tool to aid our further understanding of chemical biological processes such as phagocytosis of infectious agents.

## Supplementary Material

Supplementary information
