# Supplementary material for: Imaging of compartmentalised intracellular nitric oxide, induced during bacterial phagocytosis, using a metalloprotein–gold nanoparticle conjugate
Source: Analyst. 2017 Sep 29;142(21):4099–105. doi: 10.1039/c7an00898h (PMC5708316; doi:10.1039/c7an00898h)
Supplement: Supplementary file 1 [file AN-142-C7AN00898H-s001.pdf]

Electronic Supplementary Material (ESI) for Analyst.
This journal is © The Royal Society of Chemistry 2017

SUPPORTING INFORMATION

Imaging of Compartmentalised Intracellular Nitric Oxide, Induced during Bacterial Phagocytosis, using a Metalloprotein-Gold Nanoparticle Conjugate

Richard Leggett,[a] Paul Thomas,[b] María J. Marín,[a] Jelena Gavrilovic*,[b] and David A. Russell*,[a]

[a]School of Chemistry, [b]School of Biological Sciences, University of East Anglia, Norwich Research Park, Norwich, Norfolk, NR4 7TJ, UK.

Email: d.russell@uea.ac.uk

Email: j.gavrilovic@uea.ac.uk

EXPERIMENTAL

General

All of the reagents used were of analytical grade and used without further purification, with the exception of horse heart cytochrome *c*. All of the reagents were purchased from Sigma-Aldrich (UK) unless otherwise stated. Phosphate buffer, sodium citrate dihydrate, penicillin-streptomycin (Pen-Strep), iso-propanol, a gas tight Hamilton syringe and 30 kDa molecular weight cut-off centrifuge tubes were purchased from Fisher Scientific (UK). Cation exchange resin carboxymethyl- (CM-) cellulose, Sephadex PD 10 columns and G25 gel filtration resin were purchased from GE Healthcare (UK). 3 kDa Amicon filters were purchased from Amincon inc (USA). Alexa Fluor 488 carboxylic acid succinimidyl ester (a488), pyruvate, Leibovitz's L-15 medium (L-15), Texas Red, *S*-nitroso-*N*-acetylpenicillamine (SNAP), 250 mL Greiner culture flasks and cryogenic vials were purchased from Invitrogen (UK). *Escherichia coli* (*E. coli*) DH5α competent cells were purchased from Gibco (UK). *N*-succinimidyl 3(2-pyridyldithio)-propionate (SPDP) was purchased from Perbio Science Ltd (UK). Cell scrapers were purchased from Nalge Nunc International (UK). Carbon coated 200 mesh copper grids were purchased from Agar Scientific (UK). Anaerobic cuvettes were purchased from Hellma (Germany). The cell line RAW264.7γ NO^- was purchased from ATCC (USA). 18 mm diameter cover slips were purchased from VMR International (USA).

The UV-visible absorption spectra were recorded using a Hitachi U3000 spectrophotometer. The fluorescence emission and excitation spectra were recorded using a FluoroLog Horiba Jobin Yvon fluorescence spectrometer.

Synthesis of the SPDP-cytochrome c-a488 complex

Horse heart cytochrome *c* was purified, to remove any deamidated form of the protein, using cation exchange chromatography.[1] The amidated protein was retained on the column longer than the deamidated forms due to the large positive charge of the amidated protein. The oxidant potassium ferricyanide (10 mg) was added to horse heart cytochrome *c* (5 mL of 80 µM) to ensure that all of the protein was present in the Fe (III) form. A column (diameter 3.5 cm, length 25 cm) was packed with the cation exchange resin carboxymethyl- (CM-) cellulose and equilibrated with two column volumes of phosphate buffer (25 mM, pH 7) at 2 mL/min. The cytochrome *c* solution was loaded onto the column at 1 mL/min. The cytochrome *c* was eluted using phosphate buffer (100 mM, pH 7) and collected in 2 mL fractions. Cytochrome *c* elution was monitored by recording the absorption at 360 nm, where a small protein absorbance (less than 0.6 a.u., within the first 270 mL collected) corresponding to the deamidated forms was eluted prior to the large absorbance of the amidated cytochrome *c*. The amidated forms were pooled and concentrated by ultrafiltration using a 3 kDa Amicon filter, to give a final volume of 2.5 mL (118 mM).

To assist the binding of both Alexa Fluor 488 carboxylic acid succinimidyl ester (a488) and *N*-succinimidyl 3(2-pyridyldithio)-propionate (SPDP) succinimidyl ester group to the lysine residues of cytochrome *c*, the pH of the purified cytochrome *c* solution was adjusted by exchanging the buffer. Buffer exchange was achieved using a Sephadex™ prepacked PD 10 column. The column was equilibrated with carbonate buffer (25 mL of 100 mM, pH 8.3) prior to loading the sample of purified cytochrome *c* (2.5 mL). Purified cytochrome *c* was then eluted with carbonate buffer (3.5 mL of 100 mM, pH 8.3) completing the buffer exchange process.

The following solutions were deoxygenated and placed in an oxygen free environment (Faircrest glovebox; 0.1 ppm O_2): 0.1 mM cytochrome *c* (in carbonate buffer, 100 mM, pH 8.3), 2 mM a488 in DMSO and 10 mM SPDP in DMSO. To modify the horse heart cytochrome *c* with a488, a solution of purified cytochrome *c* (80 μM) dissolved in carbonate buffer (100 mM, pH 8.3, 0.5 mL) was reacted with a solution of a488 (100 μM) in DMSO (0.1 mL) and stirred for 1 h. The stirred solution was made up to 2.5 mL with carbonate buffer (100 mM, pH 8.3). Unbound reactants were removed using a Sephadex PD 10 column as described for the buffer exchange in the final step of the purification of cytochrome *c*.

To modify the cytochrome *c*-a488 complex with *N*-succinimidyl 3(2-pyridyldithio)-propionate (SPDP), SPDP (10 mM) in DMSO (225 μL) was added to the cytochrome *c*-a488 complex (60 μM of cytochrome *c*, 2.5 mL) and stirred for 30 min. Unbound reactants were removed using a Sephadex PD 10 column as previously described. Following removal of the unbound reactants, the solution of the SPDP-cytochrome *c*-a488complex was deoxygenated and retained in an oxygen free environment (Faircrest glovebox; 0.1 ppm O_2).

The conjugation of SPDP to cytochrome *c* was confirmed using an established method for the thiolation of proteins.[2] DL-dithiothreitol (DTT) specifically reduces the SPDP disulphide bond to give 2-thiopyridone, the oxidised form of DTT. 2-thiopyridone formation can be monitored at 343 nm (λ_{max} of 2-thiopyridone, ε_{343} = 8.08 x 10^3 M^{-1} cm^{-1})[2] to confirm the binding of SPDP to the cytochrome *c*-a488 complex. Typically, 0.1 mL of the SPDP-cytochrome *c*-a488 complex was diluted 1:10 using carbonate buffer (0.9 mL, 100 mM, pH 8.3) and the UV-visible absorption spectrum recorded. DTT (25 μL, 0.1 M) was then added to the cuvette and the UV-visible absorption spectrum recorded.

Synthesis of the NO nanobiosensor

Citrate reduced gold nanoparticles (AuNP) were prepared using a modification[3] of the Enüstün and Turkevich method.[4] Briefly, sodium citrate dihydrate (50 mg) was dissolved in water (50 mL). Hydrogen tetrachloroaurate (III) trihydrate (12.7 mg) was dissolved in water (100 mL), giving a pale yellow solution. Both solutions were heated to 60 °C, mixed and then further heated to 85 °C for 2.5 h with continuous stirring. The resultant solution had a deep red colour characteristic of citrate stabilised gold nanoparticles of 16 nm.

The fluorescently tagged SPDP-cytochrome *c* complex (35 mM protein concentration, 200 µL) was self-assembled onto the gold nanoparticles (3 nM, 20 mL) by mixing the two solutions and stirring them for 48 h. The fluorescently tagged protein-nanoparticle conjugates were then centrifuged using 30 kDa molecular weight cut-off centrifuge tubes to remove the unbound SPDP-cytochrome *c*-a488 complex. The centrifugation process was repeated a total of 3 times.

Preparation and calibration of the nitric oxide (NO) solution

Preparation of the nitric oxide solution

To determine the fluorescence intensity changes of the NO nanobiosensor when NO is bound to the cytochrome *c*, a calibrated NO solution was used. The NO solution was prepared by the addition of gaseous NO (8 mL), using a gas tight Hamilton syringe, to distilled water adjusted to pH 3 (the acidic solution allows the NO_2^- produced to be recycled to NO) with HCl (0.1 M, 3 mL) producing a *ca.* 2 mM saturated NO solution. The gaseous NO used to prepare the NO solution was achieved by bubbling pure NO through a solution of NaOH (0.1 M) that had been previously deoxygenated with N_2 for 1 h. This procedure allows the elimination of impurities such as NO_2, N_2O_3 and N_2O_4 from the NO gas.[5]

Calibration of the nitric oxide solution

To calibrate the NO saturated solution an adaptation of a known method[6] was developed in an oxygen free environment (Faircrest glovebox; 0.1 ppm O_2). A pre-packed column containing G25 gel filtration resin was equilibrated with deoxygenated phosphate buffer (25 mL of 20 mM, pH 7.4). Sodium dithionite (0.5 mL of 1 M) was added to the column to remove oxidation products. Excess sodium dithionite was flushed through the column using deoxygenated phosphate buffer (25 mL of 20 mM, pH 7.4). The redox centre of myoglobin haem was reduced (from Fe (III) to Fe (II)) by addition of sodium dithionite (5 µL, 1 M) to a solution of myoglobin (2 mL of 0.4 mM) in phosphate buffer (20 mM, pH 7.4). The reduction of the protein produced a colour change from brown to blood red. Reduced myoglobin (2 mL) was added to the G25 gel filtration column to remove the excess sodium dithionite from solution, since sodium dithionite would interfere with the NO calibration. The protein was eluted with deoxygenated phosphate buffer (20 mM, pH 7.4) and the blood red reduced myoglobin collected.

The reduced myoglobin solution (50 µL) was diluted with deoxygenated phosphate buffer (950 µL, 20 mM). The UV-visible absorption spectrum of the reduced myoglobin solution was recorded using anaerobic cuvettes. Aliquots (1 µL) of the NO saturated solution were added to the reduced myoglobin solution and the UV-visible absorption spectra recorded to determine the accurate NO concentration in solution. A calibration curve was obtained from the difference absorption spectra, plotting the absorbance difference between 436 nm and 409 nm against the volume of the NO solution added (see **Figure Si**). From the calibration curve, the reduced myoglobin solution proved to be fully bound to NO when 5 µL of the NO solution was added; hence 5 µL of NO was required to saturate 10 nmoles of reduced myoglobin. Therefore, the concentration of the NO solution was 2 mM. The reported

experiments were performed using freshly prepared solutions of NO that had been calibrated with myoglobin.

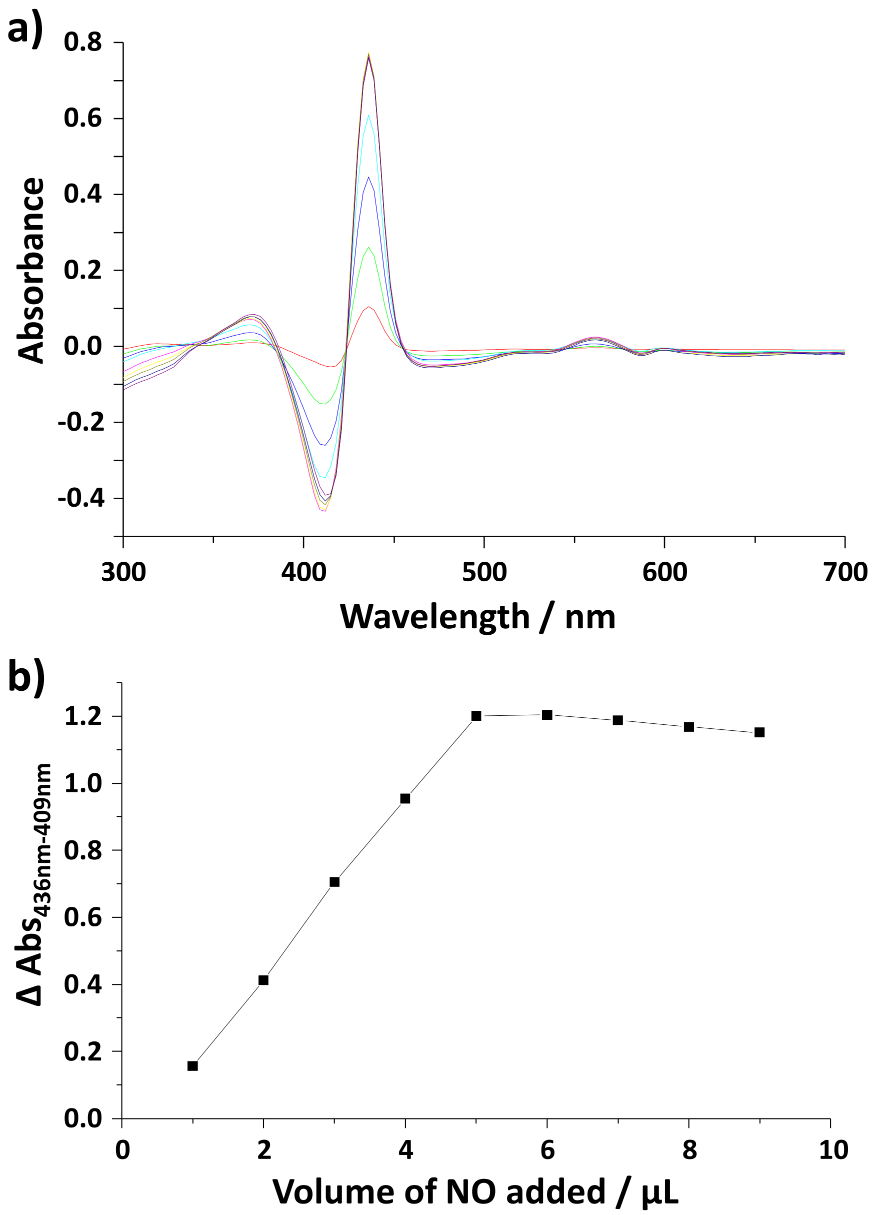

Figure Si. a) Difference absorption spectra of reduced myoglobin following addition of increasing amounts of a NO solution. **b)** Calibration curve where the absorbance difference between 436 nm and 409 nm has been plotted against the volume of NO added showing that myoglobin was fully nitrosylated upon addition of 5 µL of NO solution.

Characterisation of the NO nanobiosensor

Transmission electron microscopy

Transmission electron microscopy (TEM) was used to characterise the size and morphology of the gold nanoparticles before and after self-assembly of the modified cytochrome *c*. A 5 µL drop of the nanoparticle sample was placed onto a carbon coated 200 mesh copper grid. Excess liquid was removed first by contacting the side of the grid with adsorbent paper tissue and then allowed to dry for a further 5 min. Images were taken using a JEOL 2000EX transmission electron microscope operating at 100 kV.

Sensitivity of the NO nanobiosensor

To determine the change in fluorescence of the nanobiosensor upon binding of NO, a calibrated NO solution was used. 1 mL of a solution of the NO nanobiosensor was placed in an anaerobic fluorescence cuvette. The fluorescence emission and excitation spectra of the NO nanobiosensor were recorded before and 10 minutes following addition of increasing concentrations of the NO solution (from 0 to 300 µM). Each addition of NO was repeated in triplicate and a calibration curve of the fluorescence emission intensity at 514 nm versus the concentration of NO plotted.

Selectivity of the NO nanobiosensor

The selectivity of the NO nanobiosensor was established by challenging the nanobiosensor to a variety of interferences that potentially could be found in the macrophage cells such as low pH, other oxidising species (hydrogen peroxide, superoxide radical anion, peroxynitrite anion), nitrite and nitrate (**Table S1**). The possible interference effects of reagents used during cell culture procedures such as Penicillin-Streptomycin, lipopolysaccharide (LPS),

interferon-γ (IFN-γ), and of the cellular constituents of the RAW264.7γ NO^- macrophage cells were also studied. The interferents studied were prepared as detailed below:

pH 4-7

A solution of the NO nanobiosensor was buffered to pH values between 4 and 7 at 0.5 intervals. For pH values between 4 and 6 the NO nanobiosensor solution was citrate buffered (10 mM) and for pH values between 6.5 and 7 phosphate buffer (10 mM) was used.

Penicillin-Streptomycin (Pen-Strep), lipopolysaccharide (LPS) and interferon-γ (IFN-γ)

Pen-strep, LPS and IFN-γ, reagents required in the cell culture methods, were added to the NO nanobiosensor solution in the same concentrations as used in cell culture. The concentrations of the reagents were: 100 units/mL of Pen-Strep; 10 ng/mL of IFN-γ and 500 ng/mL of LPS; and a combination of 10 ng/mL IFN-γ with 500 ng/mL LPS.

RAW264.7γ NO^- cellular contents

To establish whether the sensitivity of the NO nanobiosensor towards NO was affected by the cellular content of the RAW264.7γ NO^- macrophages, the cells were lysed using a lysis buffer (50 mM HEPES, 150 mM NaCl, 1.5 mM $MgCl_2$, 1 mM ethylene glycol bis(2-aminoethyl ether)-N,N,N'N'-tetraacetic acid (EGTA) and 1 mM ethylenediaminetetraacetic acid (EDTA) containing 10% v/v glycerol and 1% trigene). The lysis buffer (2 mL) was added to the RAW264.7γ NO^- cells (2 mL) cultured for passaging. 200 µL of the resultant solution was added to the solution of the NO nanobiosensor.

Hydrogen peroxide (H_2O_2)

A 30% (w/w) hydrogen peroxide solution was made up to give a 0.1 M stock solution prepared in water. This solution was prepared immediately prior to use and added to the solution of the NO nanobiosensor to give the desired final concentration of 5 µM.

Superoxide anion (O_2^-)

Potassium superoxide (KO_2) solutions were used to prepare standards of superoxide anion.[7, 8] KO_2 is sparingly soluble in dry DMSO and the use of 18-crown-6 increases the solubility of KO_2 allowing pale yellow solutions to be prepared as long as the crown ether is in at least 2:1 excess of KO_2.[7] A 0.05 M solution of KO_2 was prepared in a 0.15 M crown-ether:DMSO solution and sonicated until both KO_2 and the crown ether had fully dissolved. The superoxide anion concentration was determined to be 620 µM by UV-visible absorption spectroscopy (ε_{250nm} = 2686 ± 29 M^{-1} cm^{-1}).[9] The superoxide solution was diluted to a final concentration of 5 µM using the 0.15 M crown-ether:DMSO solution.

Peroxynitrite anion ($ONOO^-$)

The production of peroxynitrite anion was achieved *via* the auto-oxidation of hydroxylamine in an alkaline solution.[10] This method is based upon the following reactions:[10]

$$NH_2O^- + O_2 \rightarrow H_2O_2 + NO^- \qquad \text{(Reaction S1)}$$

$$NO^- + O_2 \rightarrow ONOO^- \qquad \text{(Reaction S2)}$$

The reaction proceeds by the attack of oxygen on a deprotonated species yielding nitroxyl ion which is then further oxidised to peroxynitrite. In the presence of the metal chelating agent ethylene diaminetetraacetic acid (EDTA), the peroxynitrite ion is stabilised. Experiments were carried out with solutions containing 10 mM of hydroxylamine, 0.5 M of sodium hydroxide and 1 mM of EDTA. The solutions were bubbled with oxygen and stirred vigorously for 4 h. Following this, manganese (IV) oxide (20 mg) was added to the solution. The solution was filtered and stored in the freezer (-18 °C). Peroxynitrite ion concentration

was determined to be 270 µM by UV-visible absorption spectroscopy (ε_{302nm} = 1670 M^{-1} cm^{-1}).[10] The peroxynitrite solution was diluted to a final concentration of 5 µM.

Sodium nitrite

A stock solution (1 mM) of sodium nitrite was prepared in water. 5 µL or 40 µL of the nitrite stock solution was added to a solution of the NO nanobiosensor to give a final concentration of 5 or 40 µM, respectively.

Sodium nitrate

A stock solution (1 mM) of sodium nitrate was prepared in water. 5 µL or 40 µL of the nitrite stock solution was added to a solution of the NO nanobiosensor to give a final concentration of 5 or 40 µM, respectively.

Reversibility of the NO nanobiosensor for NO sensing

To determine whether the nanobiosensor could be used for multiple measurements of NO, the reversibility of the sensor was studied. Solutions of the NO nanobiosensor (1 mL) were deoxygenated and the fluorescence emission spectra recorded. NO was added to each solution to give a final concentration of 50 µM and the fluorescence emission spectra were recorded. To remove the bound NO from the cytochrome *c*, excess sodium dithionite (1 mg) was added to the solution to reduce the haem-iron (Fe (III) to Fe (II)). Potassium ferricyanide (1 mg) was then added to re-oxidise the haem-iron (Fe (II) to Fe (III)) to allow further sensing of NO. The addition of sodium dithionite and potassium ferricyanide was repeated a total of 5 times to study the reversibility of the NO nanobiosensor. Each measurement was repeated in triplicate.

RAW264.7γ NO^- cell culture

To monitor macrophage production of NO, the macrophage cell line RAW264.7γ NO^- was cultured in a humidified atmosphere of 5% CO_2 at 37 ˚C in Dulbecco's Modified Eagle's Medium (DMEM) (500 mL) containing 4.5 g/L glucose, 0.58 g/L GlutaMAX™ I, 2 mM pyruvate and supplemented with 10% fetal calf serum (FCS) and 100 units/mL of Penicillin-Streptomycin. This cell line requires both interferon-γ (IFN-γ) and lipopolysaccharide (LPS) stimulation to produce NO.[11]

Frozen aliquots of RAW264.7γ NO^- cells were taken from liquid nitrogen storage and rapidly thawed in a water bath at 37 °C for *ca.* 2 min. The cell suspension (1.5 mL) was decanted into a 15 mL centrifuge tube and resuspended in supplemented DMEM (13.5 mL), prior to centrifugation for 5 min at 25 °C and 112 xg. Waste medium was removed and the remaining pellet was resuspended in supplemented DMEM (10 mL). Resuspended cells (0.5 mL) were added to 35 mm diameter culture dishes containing supplemented DMEM (2 mL) and sterile 18 mm diameter cover slips. The cells were then incubated at 37 °C in a 5% CO_2 environment for 5 h.

Cellular experiments using the NO nanobiosensor

Following attachment of the RAW264.7γ NO^- cells to the coverslips, the medium was removed from the culture dishes, the NO nanobiosensor (10 nM) was added to the RAW264.7γ NO^- cells and the macrophages were stimulated using one of the following protocols: (A) *Lipolysaccahride and interferon-γ stimulation, t*o fully stimulate the RAW264.7γ NO^- cells to produce NO, the cells were placed in culture medium (2.5 mL) supplemented with LPS (500 ng/mL) and IFN-γ (10 ng/mL) to activate the inducible nitric oxide synthase (iNOS).[11] After addition of the supplemented culture medium to each dish containing a coverslip, the cells were further incubated at 37 °C in a 5% CO_2 environment overnight (at least 16 h); (B) *Interferon-γ stimulation,* RAW264.7γ NO^- cells were also

incubated in culture medium (2.5 mL) supplemented with IFN-γ (10 ng/mL) alone. As in (A), the cells on the coverslips were incubated at 37 °C in a 5% CO_2 environment overnight (at least 16 h); and (C) *Absence of stimulation (control),* control experiments were performed with cells that had not been stimulated to activate the production of NO. Cells were incubated overnight as in (A) and (B) but in culture medium without IFN-γ and LPS. Experiments performed with cells treated under these conditions provided the baseline NO levels.

DMEM medium is known to interfere with cell imaging as it contains riboflavins that fluoresce and phenol red which quenches fluorescence and strongly absorbs light in the red region of the electromagnetic spectrum.[12] To minimise spectral interference when imaging the RAW264.7γ NO^- cells of interest, the DMEM medium was replaced with an Imaging Medium (IM) that consisted of 120 mM NaCl, 3 mM KCl, 2 mM $CaCl_2$, 1 mM $MgCl_2$, 1 mM NaH_2PO_4, 1 mM $NaHCO_3$, 25 mM HEPES/NaOH, pH 7.2 to which 11 mM glucose, 2 mM glutamine, BME amino acids and 1 mg/mL BSA (Fract. V) were added. Following removal of the DMEM medium, the cells were washed with IM and covered with the same medium (2.5 mL). The IM used to cover the cells on the coverslip contained the appropriate concentrations of LPS and IFN-γ to ensure the correct stimulation conditions.

In some cellular experiments, to simulate the uptake of foreign bodies, RAW264.7γ NO^- cells were “challenged” with 3 µm diameter latex beads. During the media exchange process, latex beads were added (1 µL per 10 mL of Leibovitz’s L-15 medium) and incubated for 1 h at 37 °C in a 5% CO_2 atmosphere.

To conduct image analysis of the RAW264.7γ NO^- cells, the coverslip of interest was removed from the appropriate culture dish and securely tightened into a Ludin chamber

(Life Imaging Services, Reinach, Switzerland). The coverslip was washed three times with IM. The Ludin chamber was mounted on a heated stage of the microscope. Images were collected either on a Zeiss LSM510 META or a Leica TCS SP2 confocal microscope with a 63x objective (1.4 NA) and a pinhole adjusted to obtain an optical section of 1.5 µm (*ca.* 2 Airy Units). The NO nanobiosensor was excited with the 488 nm line of an argon ion laser and the fluorescence emission collected between 505 and 545 nm. Corresponding differential interference contrast (DIC) images were generally obtained using the 488 nm laser line, occasionally with the 543 nm HeNe laser.

N_ω-nitro-L-arginine methyl ester hydrochloride (L-NAME) is a known inhibitor of iNOS,[13, 14] and consequently will prevent the production of NO. RAW264.7γ NO^- cells were treated overnight with the NO nanobiosensor and stimulated with medium containing both LPS and INF-γ that had been further supplemented with L-NAME (100 µM). Following medium exchange, fluorescence images of the treated cells were taken as indicated previously for RAW264.7γ NO^- cells that had been incubated with the NO nanobiosensor under different stimulation conditions.

To confirm that the NO nanobiosensor was responsive to NO within the intracellular environment, a NO donor was added to the RAW264.7γ NO^- cells. The NO donor used was *S*-nitroso-*N*-acetylpenicillamine (SNAP), which spontaneously produces NO under physiological conditions.[15, 16] After overnight incubation with the NO nanobiosensor and stimulation with either INF-γ, LPS and INF-γ, or non-stimulation, the medium was exchanged and the cells were treated with SNAP (100 µM). The cells were incubated for 40 min prior to confocal imaging following the conditions described for RAW264.7γ NO^- cells that had been incubated with the NO nanobiosensor.

The fluorescence emission intensity of the NO nanobiosensor from within the intracellular environment was analysed in confocal images by first removing dead cells and cells at the edges of the images that were not completely in the field of view. Then the DIC images were used to obtain a count of the remaining cells. The fluorescent spots were then counted and their size and fluorescence emission intensity measured using a custom-written macro in Fiji (ImageJ) which combined background subtraction (rolling-ball), thresholding (Huang algorithm) and separation of touching objects (Watershed). The data were finally normalised for each image by dividing by the number of cells in the image.

Extracellular measurements of NO using the NO nanobiosensor

The nanobiosensor was used to determine the amount of NO produced by RAW264.7γ NO^- cells in the extracellular environment. These results were compared to those obtained using a known electrochemical method.[17] The ISO-NOP™ Mark II NO electrode (ISO-NOP) (World Precision Instruments, UK) is an amperometric sensor covered by a gas permeable hydrophobic membrane that allows NO to penetrate through and be oxidised on the working electrode.[18] The oxidation creates a current the magnitude of which can be related directly to the concentration of NO in the sample. The current is related to the potential applied to the electrode, which results from the oxidation of NO, which in turn is dependent directly on the rate of delivery, or rate of diffusion, of NO to the electrode surface. The ISO-NOP has good selectivity as the electrodes are separated from the sample in which the measurements are being made by gas-permeable hydrophobic membranes, ruling out any interference from solutions or dissolved species other than gases. The membrane is permeable to all gases. The selectivity of the sensor for NO over other gases which permeate the membrane and coatings is determined by the potential which is applied to the electrode. Of the biologically relevant gases which have been tested using the ISO-NOP only CO_2 is known to interfere, however at physiological pH, the concentration of CO_2 is relatively

constant and so any signal due to dissolved CO_2 can be included in the baseline measurement. Therefore, the ISO-NOP can be used to make direct measurement of NO within the extracellular environment and this measurement can be compared with that obtained from the NO nanobiosensor.

RAW264.7γ NO^- cells were cultured and stimulated overnight with LPS and INF-γ. The ISO-NOP was calibrated by adding aliquots (1 µL) of the NO solution (2 mM) to 2 mL of deoxygenated phosphate buffer (20 mM, pH 7.4), which contained the ISO-NOP electrode. The supernatant above the cultured and stimulated RAW264.7γ NO^- macrophage cells contained some RAW264.7γ NO^- cells, which were not stuck to the coverslip together with any NO produced by the cells. Aliquots of the supernatant above the cultured and stimulated RAW264.7γ NO^- cells (1 µL) were then added to a fresh 2 mL of deoxygenated phosphate buffer (20 mM, pH 7.4) containing the ISO-NOP electrode and the electrochemical measurement of NO recorded. For the NO nanobiosensor, extracellular contents (300 µL) were added to a solution of the deoxygenated NO nanobiosensor (1 mL) and the fluorescence emission spectra recorded. The increase in fluorescence upon addition of the supernatant was then converted to a concentration of NO based on the calibration curve obtained for the NO nanobiosensor used, considering the dilution effect. All the measurements were recorded in triplicate of five repeats of each stimulation condition.

Intracellular experiments with the NO nanobiosensor and Escherichia coli DH5α bacteria

To challenge RAW264.7γ NO^- cells with a natural stimulus, *Escherichia coli* (*E. coli*) DH5α bacteria were used. To enable fluorescence imaging, the bacteria were labelled with a red fluorophore, Texas Red, as follows. 1% formalin fixed *E. coli* DH5α competent cells (1 mL) were thawed and centrifuged at 1006 xg at 25 °C for 4 min (ALC refrigerated centrifuge) to produce a small pellet. The supernatant was removed and the pellet was resuspended in

sterile water (1 mL). The centrifugation and resuspension procedure was repeated a total of three times to ensure all fixative was removed. Texas Red (2 µL of 1 mg/mL) was added to the bacterial suspension where penetration of the fluorophore into the bacteria was facilitated by gentle pipetting. The Texas Red bacterial solution was incubated in the dark at 4 ˚C for 18 h. The fluorescently modified *E. coli* were then washed with sterile water (1 mL) by centrifugation and resuspension. This centrifugation/resuspension procedure was repeated a total of 10 times to ensure all free fluorophore was removed. The bacteria were stored (no longer than 7 days) at 4 ˚C.

RAW264.7γ NO^- cells were incubated overnight with the NO nanobiosensor and stimulated with LPS and INF-γ as described previously. 3 h prior to imaging with the confocal microscope, fluorescently labelled bacteria (10 µL) were added and incubated at 37 °C in a 5% CO_2 environment. Fluorescence images of RAW264.7γ NO^- cells incubated with *E. coli* DH5α were obtained using a Zeiss LSM 510 confocal microscope. To image the Texas Red labelled bacteria a 543 nm HeNe laser was used as the excitation source and the fluorescence emission was collected between 560 and 750 nm.

To observe the production of NO by the RAW264.7γ NO^- cells, several fluorescence images were taken over time, forming a time-lapse sequence. RAW264.7γ NO^- cells were stimulated with both LPS and INF-γ and incubated with the NO nanobiosensor overnight. Texas Red stained bacteria were added for 3 h after overnight stimulation. For time-lapse analysis, cells were imaged in the presence of 10 µM astaxanthin, a free-radical scavenger used to prevent photodamage that was added 30 min before start of imaging.[19] In the absence of astaxanthin the cells died after capture of less than 10 images. The uptake of bacteria and production of NO was monitored using the time series function on the Zeiss LSM 510 confocal microscope. The coverslip of interest was removed from the appropriate culture

dish, securely tightened into a Ludin chamber and washed three times with imaging medium. The Ludin chamber was mounted on a heated stage of the microscope. Images were collected with a 63x objective (1.4 NA) and a pinhole adjusted to obtain an optical section of 1.5 µm (*ca.* 2 Airy Units). A bacterium of interest was focused on, the Z-position marked as zero (ensuring the same optical slice was taking during the time series), and then images were obtained every 2 min as described previously, with occasional interruptions to refocus. To image the NO nanobiosensor and Texas Red bacterium simultaneously, and to ensure that the fluorescence from the NO nanobiosensor did not 'bleed' through into the bacterium signal, each image was captured using the 'line by line' function of the Zeiss LSM 510 confocal microscope. The NO nanobiosensor was excited with the 488 nm line of an argon ion laser and the fluorescence emission collected between 505 and 545 nm. To image the Texas Red labelled bacteria, a 543 nm HeNe laser was used as the excitation source and the fluorescence emission was collected between 560 and 750 nm. Corresponding differential interference contrast images were obtained using the 488 nm laser line. For analysis, a bacterium of interest was identified and then tracked manually using iQ software (Andor, Belfast, UK). In each frame, the bacterium was outlined and the mean fluorescence intensity in each channel was measured and divided by the area of the bacterium to obtain the fluorescence/μm^2.

Image processing

Adobe Photoshop 7.0 or Andor IQ were used for image processing and producing composite images of up to three fluorescence channels and differential interference contrast (DIC) images. The multi-channel fluorescence images were ordered into different channels of a red-green-blue (RGB) image in Photoshop or Andor IQ. The DIC image was converted to grey scale, blended and flattened with the RGB to create a composite image using the colour blending option of Photoshop.

SUPPORTING FIGURES

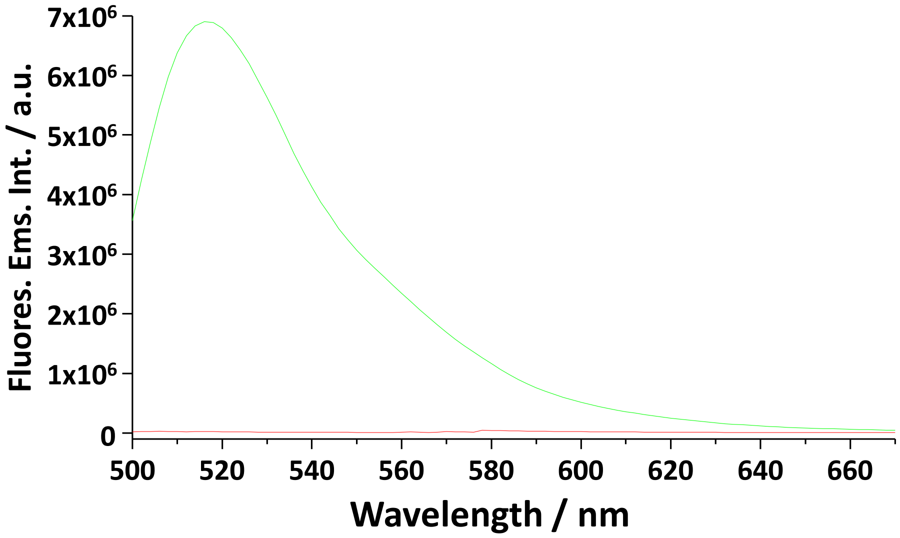

Fig. S1. Fluorescence emission spectra (λ_{exc} = 488 nm) showing the increase in fluorescence when Alexa Fluor 488 was bound to cytochrome *c* (green) (100 µM) compared with the intrinsic fluorescence of cytochrome *c* protein (red).

Table S1. Summary of interferents investigated to determine the selectivity of the NO nanobiosensor, showing the concentrations studied and the concentrations typically present within macrophages. The remaining interferents studied are not naturally present within macrophages, however the concentrations used of these were Penicillin-Streptomycin (100 units/mL), INF-γ (10 ng/mL), LPS (500 ng/mL) and a combination of INF-γ (10 ng/mL) and LPS (500 ng/mL).

Interferent	Concentration added	Concentration present within macrophage cells	Reference
**Nitrate (NO_3^-)**	5 and 40 µM	2-10 µM	[20]
**Nitrite (NO_2^-)**	5 and 40 µM	2-10 µM	[20]
**Peroxynitrite ($ONOO^-$)**	5 µM	440 nM/10^6 cells per min	[21]
**Superoxide (O_2^-)**	5 µM	100 nM/10^7 cells over 24 h	[22]
**Hydrogen peroxide (H_2O_2)**	5 µM	594 ± 94 nmol/mg of protein per h	[23]
**RAW264.7γ NO^- cellular content**	n/a	n/a	-
**pH**	4-7	Phagolysome 4.0-5.5 Cytosol 7.2-7.4	[24, 25]

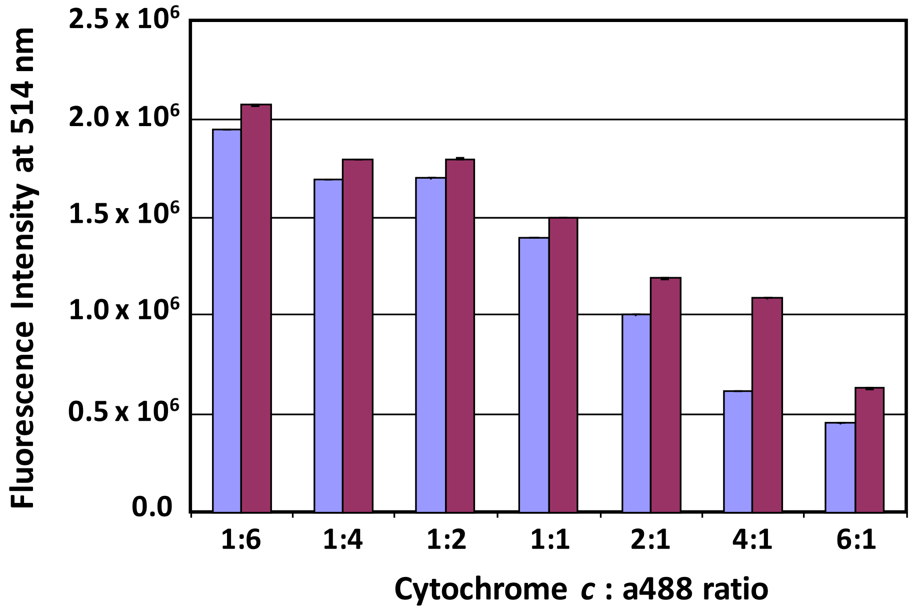

Fig. S2. Optimisation of cytochrome *c*-a488 to give the greatest change in fluorescence intensity when ■ 0 µM NO or ■ 230 µM NO was bound to the cytochrome *c*-a488 complex. Error bars show the mean error in the triplicate measurements. An optimal molar ratio of 4:1 (cytochrome *c*:a488) was found to give the greatest enhancement of fluorescent intensity in the presence of NO.

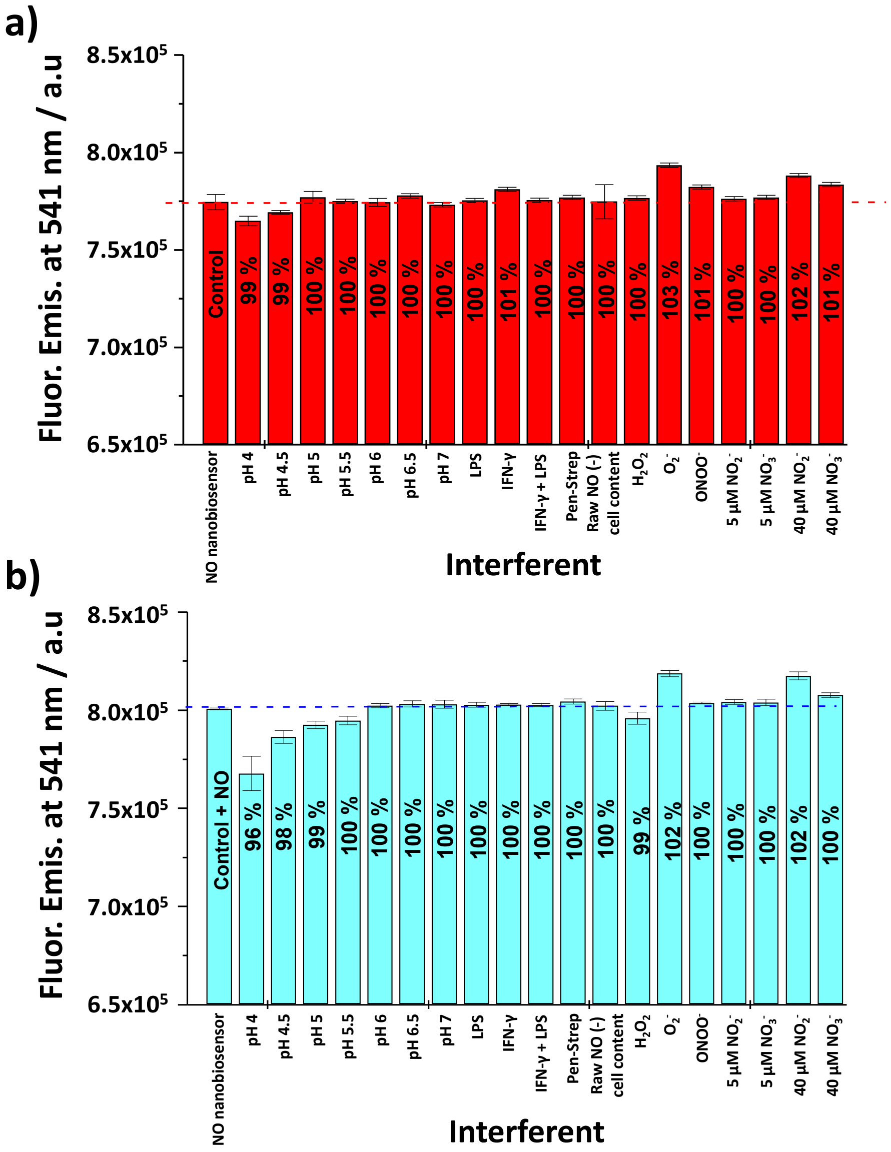

Fig. S3. Study of the selectivity of the NO nanobiosensor. Effect of the different interferents on the fluorescence emission intensity of the NO nanobiosensor: **a)** in the absence and **b)** in the presence of NO (40 µM).

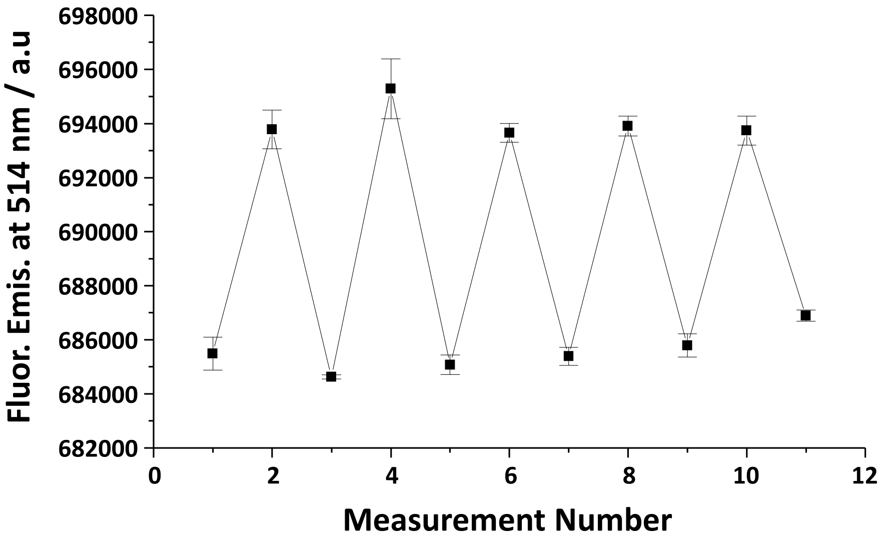

Fig. S4. Reversibility of the NO nanobiosensor towards NO. Odd numbered measurements were recorded in the absence of NO and even numbered measurements were taken after the addition of NO (50 µM).

Table S2. Statistical analysis of the fluorescence emission intensities of unstimulated control cells, cells stimulated with IFN-γ only, stimulated both with IFN-γ and LPS and cells stimulated with both IFN-γ and LPS in the presence of L-NAME.

**Sample**	Area of fluorescence per cell (Mean ± SEM)	Fluorescence intensity (Mean ± SEM)	Integrated fluorescence intensity per cell (Mean ± SEM)
**Controls [n = 14]**	8.2 ± 0.95 μm^2	34.7 ± 1.53 AU	292 ± 41.7 AU·μm^2
**IFN-γ [n = 13]**	6..7 ± 1.02 μm^2	31.8 ± 2.83 AU	234 ± 56.6 AU·μm^2
**IFN-γ+LPS [n = 15]**	42.3 ± 3.64 μm^2 [a,b,c]	56.1 ± 3.03 AU [d,e,f]	2447 ± 286.8 AU·μm^2 [g,h,i]
**IFN-γ+LPS+L-NAME [n = 5]**	8.5 ± 1.51 μm^2	40.4 ± 2.09 AU	341 ± 67.1 AU·μm^2

Mean: Mean of the measurement; SEM: Standard Error of the Mean
[a,d,g] Significantly different from Controls ($p < 0.001$)
[b,e,h] Significantly different from IFN-γ ($p < 0.001$)
[c,i] Significantly different from IFN-γ+LPS+L-NAME ($p < 0.001$)
[f] Significantly different from IFN-γ+LPS+L-NAME ($p < 0.05$)
Controls, IFN-γ and IFN-γ+LPS+L-NAME were not significantly different from each other.
Comparisons were made by one-way ANOVA followed by post t-test with Bonferroni correction for multiple comparisons.
n is the number of images analysed each containing on average 12 cells.

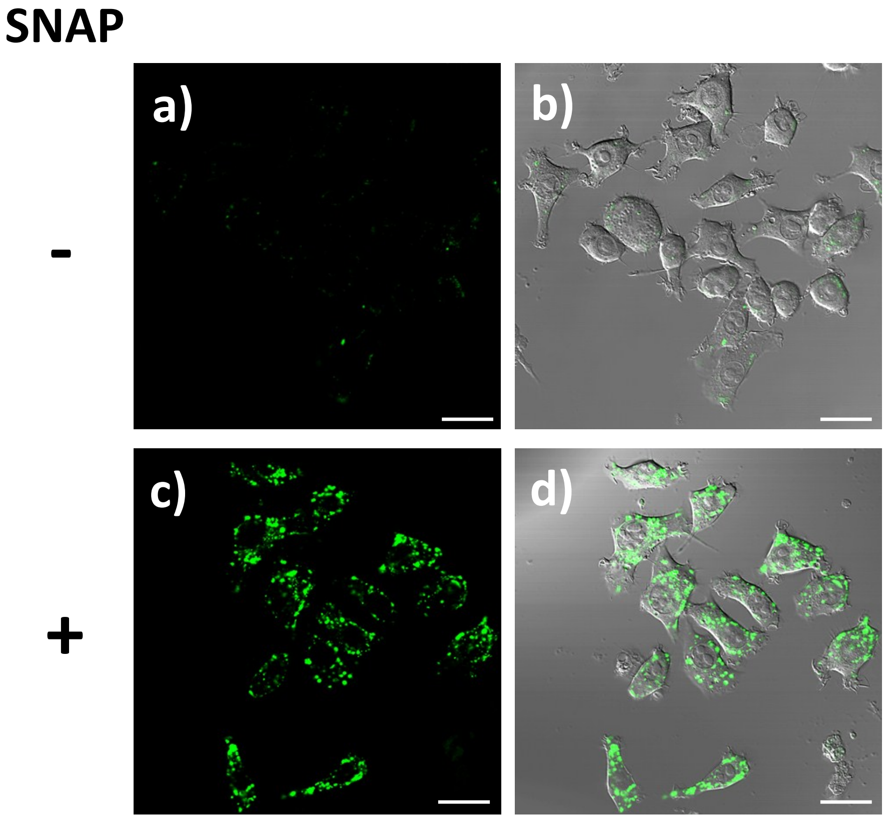

Fig. S5. Confocal fluorescence microscopy images of RAW264.7γ NO^- cells incubated overnight with the NO nanobiosensor (10 nM) and INF-γ (10 ng/mL) and **a** and **b)** no SNAP added or **c** and **d)** incubated with SNAP (100 µM) 40 min prior to imaging. Images **a** and **c** are fluorescence images obtained in the green channel (505 – 545 nm; λ_{ex} = 488 nm). Images **b** and **d** are composite images of the green and differential interference contrast (DIC) channels. Scale bars = 20 µm.

Table S3. Statistical analysis of the fluorescence emission intensities of unstimulated control cells, cells stimulated with IFN-γ only and cells stimulated both with IFN-γ and LPS non-incubated and incubated with the NO donor SNAP.

**Sample**	Area of fluorescence per cell (Mean ± SEM)	Fluorescence intensity (Mean ± SEM)	Integrated fluorescence intensity per cell (Mean ± SEM)
**Controls [n = 6]**	6.6 ± 0.59 μm^2	24.5 ± 1.36 AU	166 ± 21.9 AU·μm^2
**Controls+SNAP [n = 6]**	36.4 ± 5.84 μm^2 [a]	50.1 ± 2.00 AU [b]	1850 ± 326.4 AU·μm^2 [c]
**IFN-γ [n = 6]**	9.8 ± 1.23 μm^2	24.8 ± 1.56 AU	249 ± 44.5 AU·μm^2
**IFN-γ+SNAP [n = 6]**	73.0 ± 7.22 μm^2 [d]	60.3 ± 1.61 AU [e]	4440 ± 518.6 AU·μm^2 [f]
**IFN-γ+LPS [n = 6]**	69.7 ± 6.59 μm^2	51.4 ± 3.61 AU	3441 ± 337.8 AU·μm^2
**IFN-γ+LPS+SNAP [n = 6]**	72.7 ± 7.70 μm^2 [g]	48.1 ± 2.06 AU [h]	3479 ± 340.2 AU·μm^2 [i]

Mean: Mean of the measurement; SEM: Standard Error of the Mean
[a] Significantly different from Controls ($p < 0.001$)
[b,c] Significantly different from Controls ($p < 0.01$)
[d,e,f] Significantly different from IFN-γ ($p < 0.001$)
[g,h,i] Not significantly different from IFN-γ+LPS ($p > 0.05$)
Comparisons were made between samples with and without SNAP using a one-way ANOVA followed by post t-test with Bonferroni correction for multiple comparisons.
n is the number of images analysed each containing on average 18 cells.

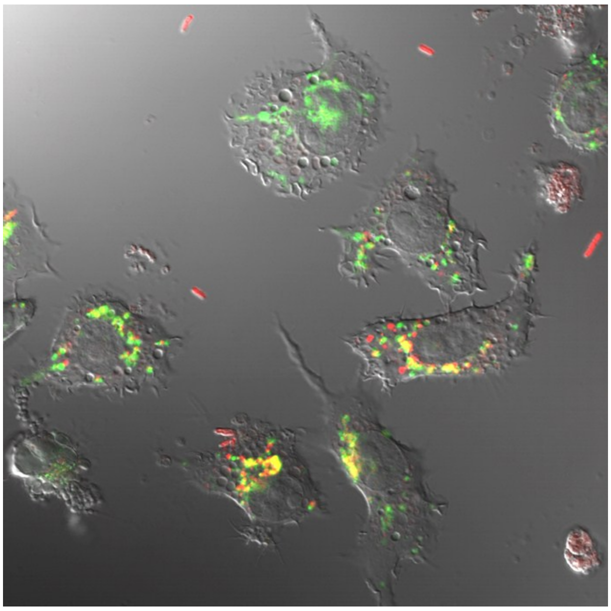

Fig. S6. Confocal fluorescence microscopy image of RAW264.7γ NO^- macrophage cells stimulated with IFN-γ (10 ng/mL) and LPS (500 ng/mL) and incubated with the NO nanobiosensor (10 nM) overnight; and then challenged with Texas Red stained *E. coli* bacteria for 3 h prior to imaging. The green fluorescence is due to the emission of the NO nanobiosensor upon excitation at 488 nm (emission collected between 505 – 545 nm), the red emission is due to either intact or fragments of Texas Red-tagged *E. coli* (λ_{exc} = 543 nm, emission collected between 560 – 750 nm) and the yellow areas are the co-localised NO nanobiosensor and Texas Red-tagged *E. coli.* The figure is a composite image of the green, red and DIC channels.

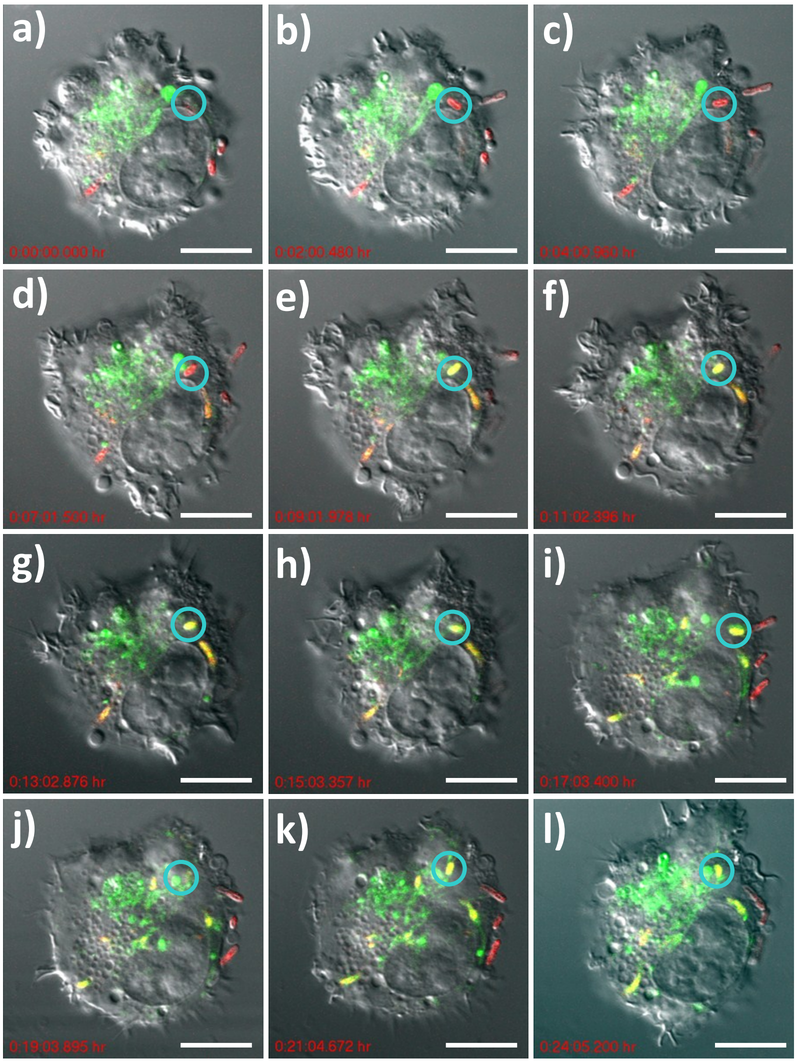

Fig. S7. Time course of NO generation during bacterial phagocytosis. Fluorescence images obtained every 2 min using time-lapse confocal microscopy of RAW264.7γ NO^{-} macrophage cells stimulated with IFN-γ (10 ng/mL) and LPS (500 ng/mL) and incubated with the NO nanobiosensor (10 nM) overnight; and then challenged with Texas Red-stained *E. coli* bacteria for 3 h prior to imaging. The green fluorescence is due to the emission of the NO nanobiosensor (λ_{exc} = 488 nm, emission 505 – 545 nm), the red emission is due to either intact or fragments of Texas Red-tagged *E. coli* (λ_{exc} = 543 nm, emission 560 – 750 nm) and the yellow areas are the co-localised NO nanobiosensor and Texas Red-tagged *E. coli.* Images **a)** – **l)** are composite images of the green, red and DIC channels. Cyan circle shows an example of the co-localisation of the bacterium with the NO nanobiosensor. Scale bars = 10 µm.

REFERENCES

1. D. L. Brautigan, S. Ferguson-Miller and E. Margoliash, in *Methods in Enzymology*, eds. S. Fleischer and L. Parker, Academic Press, 1978, pp. 128-164.
2. T. Y. Imoto, H. , in *Protein Function - A Practical Approach*, ed. T. E. Creighton, IRL Press, Oxford, 1997, pp. 247-259.
3. D. C. Hone, A. H. Haines and D. A. Russell, *Langmuir*, 2003, **19**, 7141-7144.
4. B. V. Enüstün and J. Turkevich, *J. Am. Chem. Soc.*, 1963, **85**, 3317-3328.
5. M. D. Lim, I. M. Lorković and P. C. Ford, in *Methods in Enzymology*, Academic Press, 2005, vol. 396, pp. 3-17.
6. M. S. Feelisch and J. S. Stamler, in *Methods in Nitric Oxide Research*, John Wiley & Sons, Chichester, 1997, pp. 455-478.
7. J. S. Valentine and A. B. Curtis, *J. Am. Chem. Soc.*, 1975, **97**, 224-226.
8. B. R. Lokesh and M. L. Cunningham, *Toxicol. Lett.*, 1986, **34**, 75-84.
9. S. Kim, R. DiCosimo and J. San Filippo, *Anal. Chem.*, 1979, **51**, 679-681.
10. X.-F. Yang, X.-Q. Guo and Y.-B. Zhao, *Talanta*, 2002, **57**, 883-890.
11. W. J. Murphy, M. Muroi, C. X. Zhang, T. Suzuki and S. W. Russell, *J. Endoxtin Res.*, 1996, **3**, 381-393.
12. S. Hollingworth and S. M. Baylor, *J. Gen. Physiol.*, 1990, **96**, 473-491.
13. T. B. McCall, M. Feelisch, R. M. J. Palmer and S. Moncada, *Br. J. Pharmacol.*, 1991, **102**, 234-238.
14. O. M. S. Abdallahi, H. Bensalem, M. Diagana, M. De Reggi and B. Gharib, *Parasitology*, 2001, **122**, 309-315.
15. G. Müller-Strahl, K. Kottenberg, H.-G. Zimmer, E. Noack and G. Kojda, *J. Physiol.*, 2000, **522**, 311-320.
16. R. P. Haugland, in *Handbook of Fluorescent Probes and Research Products* Molecular Probes Inc, Eugene, 9th edn., 2002, pp. 128-164.
17. U. Simonsen, R. M. Wadsworth, N. H. Buus and M. J. Mulvany, *J. Physiol.*, 1999, **516 (Pt 1)**, 271-282.
18. G.-W. He and Z.-G. Liu, *Circulation*, 2001, **104**, I-344-I-349.
19. L. Sacconi, D. A. Dombeck and W. W. Webb, *Proc. Natl. Acad. Sci. U. S. A.*, 2006, **103**, 3124-3129.
20. B. Chen and W. M. Deen, *Chem. Res. Toxicol.*, 2002, **15**, 490-496.
21. H. Ischiropoulos, L. Zhu and J. S. Beckman, *Arch. Biochem. Biophys.*, 1992, **298**, 446-451.
22. Y. Xia and J. L. Zweier, *Proc. Natl. Acad. Sci. U.S.A.*, 1997, **94**, 6954-6958.
23. A. H. Ding, C. F. Nathan and D. J. Stuehr, *J. Immunol.*, 1988, **141**, 2407-2412.
24. M. J. Marín, F. Galindo, P. Thomas and D. A. Russell, *Angew. Chem., Int. Ed.*, 2012, **51**, 9657-9661.

25. F. Galindo, M. I. Burguete, L. Vigara, S. V. Luis, N. Kabir, J. Gavrilovic and D. A. Russell, *Angew. Chem., Int. Ed.*, 2005, **44**, 6504-6508.
